# Supplementary material for: Are carbon emissions trading and green financial instruments synergistic? -Comprehensive quantitative research based on content analysis
Source: PLoS One. 2024 Mar 7;19(3):e0298601. doi: 10.1371/journal.pone.0298601 (PMC10919601; doi:10.1371/journal.pone.0298601)
Supplement: S1 Data — (ZIP) [file pone.0298601.s001.zip › Data/地级市Z_F工作报告.zip!/║┌┴·╜¡/╣■╢√▒⌡╩╨/└┤╫╘╣1⁄2╓┌║┼í╛┬φ┐╦╩2╛▌═°í┐_2005.docx]

各位代表：

　　现在，我代表市人民政府向大会作工作报告，请予审议，并请市政协各位委员提出意见。

　　一、实施老工业基地振兴战略取得良好开局，全市经济和社会事业步入良性发展轨道

　　过去的一年，是我市全面实施老工业基地振兴战略并取得初步成效的一年。在省委、省政府和市委领导下，在市人大、市政协的监督支持下，我们以邓小平理论和“三个代表”重要思想为指导，贯彻党的十六届三中、四中全会精神，落实科学发展观，围绕“努力快发展，全面建小康，振兴哈尔滨”的工作主题，紧紧依靠全市人民，以实施振兴老工业基地战略为契机，加快结构调整，推进体制创新，扩大对外开放，经济社会发展出现了新局面。全市实现生产总值1680.5亿元，增长14.7%，增幅创1996年以来新高，其中第一、二、三产业分别增长10.5%、19.9%、12.1%。财政一般预算收入161.4亿元，增长 19.3%，其中地方财政一般预算收入95.6亿元，增长25.2%。完成固定资产投资532.6亿元，增长22.2%。社会消费品零售总额707.4亿元，增长13.3%。进出口贸易总值 21亿美元，增长10%。城市居民人均可支配收入8940元，增长13.1%。农民人均纯收入3623元，增长23.1%。

　　（一）抢抓机遇，加大改革、改造力度，工业经济速度和效益同步增长

　　在振兴老工业基地战略推动下，坚持深化改革，调整结构，转变增长方式，工业经济呈现良好发展局面。实现增加值516.6亿元，增长20.2％。经济效益综合指数继续攀升，达到115.1。实现利税80.8亿元，增长16.4%；利润24.9亿元，增长25.8％。

　　积极采取与国家政策互动的措施，努力争取中央和省的支持。对国有企业进行全方位调查，完成82户国有企业清产核资和74户企业资产评估。出台了《关于推进国有企业改革工作的实施意见》等30多项政策性文件。在国家8个方面的支持政策中，我市争取到国债项目72个，总投资144.3亿元，获得国家和省支持资金6.05亿元；争取到国家“并轨”资金4亿元，做实养老保险个人账户资金1.9亿元；争取到国家专项补贴10.5亿元。启动608户不良贷款企业的整体打包回购，完成了45所企办学校、17个企办公安机构和6所企办医院分离工作。

　　国有企业改革取得阶段性成果。制定了《关于市属国有改革改制企业职工劳动关系处理和社会保险关系接续的意见》、《国有企业改制工作程序》等规定，全市共完成290户国有企业改制，其中，重点企业63户，引入战略投资超过20亿元，转让国有资产收益15.8亿元。哈啤集团国有股转让、哈药集团增资扩股等重点企业的成功改制，为深化国有企业改革起到示范作用。积极支持中省直国有企业改制，哈航集团等企业改制取得重大进展。确定了国有资产三级管理模式，国有资产管理体制进一步完善。

　　工业结构调整取得较大进展。完成工业固定资产投资117.8亿元，增长20.1％。其中，机械制造、高新技术、食品和医药工业完成投资96.3亿元，占全市工业的81.7%。产业集群建设步伐加快，主导产业链条继续延伸。汽车工业园新开工18个配套项目；医药工业园引进企业10户；电站设备配套企业达到200户，完成配套产值5.6亿元，增长106%；食品工业实现增加值53.1亿元，增长26.8%；化工产业规划12个项目，总投资82.7亿元。

　　（二）落实惠农政策，加快结构调整，县域经济发展呈现新局面

　　发挥国家政策效能，围绕农民和县（市）财政双增收的目标，以粮牧“主辅换位”为重点，推进结构调整；以税费改革为重点，深化农村改革；以推广农村新型合作经济组织为重点，推动经营体制创新；以园区建设为重点，推动县域工业发展和产业化经营，取得了粮食增产、农业增效、农民增收、县域经济加快发展的喜人局面。实现农业增加值275.6亿元，增长10.5%。粮食总产量达到88.15亿公斤。特色、优质、高效农业发展迅速，绿色食品种植面积274.3万亩，增长94.3%。优质粮食作物面积达到2100万亩，优质率进一步提高。推进粮牧“主辅换位”，优化农业结构取得历史性突破，畜牧业和渔业总产值188.4亿元，首次超过种植业，实现了“半壁江山”的目标。推动农产品流通体系建设，积极发展订单农业。农业产业化经营快速发展，参与农户达到59万户，龙头企业发展到604户，其中产值超千万元的65户，超10亿元的3户。县域工业增势强劲。乡村道路和农田水利等基础设施建设投资8亿元，增长19.4%。培训农民工23.3万人，转移农村剩余劳动力98.17万人，占全市农村劳动力总数的42.85%。农村税费改革成果得到巩固，农村信用社改革全面启动，农村新型合作经济组织迅速兴起。

　　（三）突出重点，引导消费，服务业继续快速发展

　　继续大力发展旅游业，开放式发展商业零售业，推动和引导消费升级。第三产业实现增加值761.9亿元，增长12.1%。旅游业实现总收入102.3亿元，增长18%；接待国内外游客1533.4万人次，增长12.8%，创汇8467.7万美元，增长39.6%。冰雪旅游继续升温，欧陆风情游、金源文化游、生态游等四季旅游渐成热点。零售业继续向国内外开放，推动了商业业态更新和新商圈的形成，全市连锁商业机构发展到30家，大型超市12家。国有商业企业改革取得阶段性成果。金融环境得到改善，市商业银行改制重组工作取得新进展，银行、保险等26家外埠金融机构进驻我市。居民消费结构继续发生变化，汽车、房地产、旅游、教育的消费支出进一步提高。物流、会展、咨询、信息、交通、房地产、中介等服务业稳步发展。

　　（四）推进城市建设、管理重心下移，城市面貌发生新变化

　　在城市建设、管理中，实行市区结合、区企联动，全面引入市场机制。完成城市建设总投资170.4亿元，一批对城市长远发展和人民生活产生深远影响的项目建设取得重大进展。新一轮城市总体规划修编完成。启动1小时都市经济圈规划，完善了松北、哈西、老太平、群力地区的分区规划。松北开发进入新阶段。实施城区供热、供水、排水“三大管网”改造工程，改造面积和长度皆为历年之最。完成道里集中供热一期等工程，全市新增集中供热面积970万平方米，城市中心区冬季大气环境质量明显改善。完成向阳生活垃圾无害化处理工程、太平污水处理厂主体工程。磨盘山供水工程实现水库大坝成功截流。沿江污水截流工程完成工程量的95%。启动松北集中供热、医疗垃圾无害化处理、西南部垃圾处理场工程。新建、改造道路57条，立交桥2座。对城区120条街路、24座桥梁进行维修改造，修复路面42.8万平方米。城区植树156万株，新增绿地面积652.3公顷，绿化覆盖率达到32.1%。实施太阳岛二期改造工程。建成国内规模最大的北方森林动物园。郊区实施绿色通道、村屯绿化、两荒治理和生态圈建设四项工程，植树造林1099公顷。治理大气污染源2300个。注重汇集多样文化，完成中央大街环境综合整治三期、犹太会堂及周边环境整治、沿江风景长廊二期、果戈里大街二期改造、印度风情街等特色街路建设和改造工程，建成动力巴黎广场、道外清真寺广场、维也纳音乐广场。环境综合整治继续向贴近百姓生活领域延伸。改造一批背街巷道、城区土路、人行道、无灯街路、居民庭院，建成17个特色社区广场。集中整治地下设施检查井1.15万个。新建一批人行天桥、公共厕所和垃圾转运间。继续实施“畅通工程”，开展车辆超载专项治理。新增和更新一批公交车辆。推进城市管理体制改革，继续向各区下放管理权，城市管理重心逐步向区级转移。经营城市有新进展。严格按照国家要求，通过招拍挂形式，规范土地经营工作。对城市供热、清冰雪、环卫、绿化等公用事业进行市场化改革。采取BOT方式，建成一批基础设施项目。城建决策方式更加科学化、民主化。

　　（五）开发区牵动作用增强，对外开放取得新进展

　　坚持实施开发区牵动战略，实行“一区多园”等创新方式，开发区带动招商引资和经济发展的作用明显增强。各级各类开发区实际利用外资1.68亿美元，占全市的65.4%。以哈尔滨国家级开发区为龙头，利民、宾西、双城等开发区实现快速发展。深入开展“招商引资年”活动，哈洽会、“长三角”招商会、香港活动周等重大招商活动取得良好效果。全市直接利用外资实际投入2.57亿美元，增长13.7％。新批投资额500万美元以上项目29个，加拿大美康、美国加吉、德国麦德龙、瑞士伊文达等跨国企业进驻我市。国内经济合作取得新进展，到位资金100.6亿元，增长157.2%。对俄贸易额增长62.82%。与俄罗斯40多个科研单位建立合作关系，33个项目列入政府间合作计划，液流发生器、多用途轻型飞机等一批项目开始走向产业化。

　　（六）人民生活进一步改善，社会事业全面进步

　　坚持以人为本的行政理念，努力使人民群众在老工业基地振兴中受益。年初确定的20件实事，除轨道交通项目外全部得到落实。

　　就业和社会保障工作取得新成效。安置就业再就业12.95万人，完成14.2万名国有企业下岗职工基本生活保障向失业保险并轨。新增基本养老保险参保人员3.09万人，离退休人员基本养老金社会化发放率100%，启动实施新的养老金计发办法，做实个人账户资金筹集到位。建立失业保险市级调剂金制度，失业保险新增参保职工5.88万人。完善基本医疗保险、职工工伤和生育保险制度，扩大困难企业和灵活就业人员参加医疗保险范围，城镇医疗保险参保人数达到143万，1.5万名困难企业职工参加住院医疗统筹，1.9万名灵活就业人员得到医疗保障。

　　扶贫减贫工作取得成效。城市25.9万人享受最低生活保障，支付低保资金1.83亿元。启动农村低保试点工作。深入开展低保对象医疗救助工作，11.8万名低保对象享受基本医疗救助。深入实施“慈善济困民德工程”，接受捐款1491万元。改造危房棚户区120.8万平方米。为特困家庭解决廉租住房485套。实行供暖补贴、教育助学、慈善超市等专项救助办法。

　　事业单位改革取得突破。通过结构与布局调整，全面整合事业资源，合并、撤销和转企事业单位1220个，精简事业编制1.45万个。立足于“养事不养人”，改革财政供给方式，全面推行聘用合同制和后勤服务社会化。

　　科技、教育、文化、卫生、体育等事业取得新进展。深入实施“科教兴市”战略，以企业为主体的科技创新体系建设步伐加快，科技对经济发展的拉动作用进一步增强。实现高新技术产业产值560亿元，增长36.6%；专利申请量达到2827件，增长32%；创业投资机构发展到12家，资金规模达到20亿元。哈工大牵头自主研制的“实验卫星一号”发射成功，为国防科技建设做出重要贡献。健全人才工作机制，改善招才引智环境，吸引海内外创业者5000余人。加强教育基础工作，改扩建一批优质高中，改造80所农村薄弱学校，安排5万名农民工子女入学。文化事业异彩纷呈，成功举办了第27届哈尔滨之夏音乐会，广泛开展群众文化活动。卫生改革与发展取得新成果，人民群众的健康水平进一步提高。完成公共卫生体系建设项目27个，对23所乡镇卫生院实施危房改造，应对突发性公共卫生事件的能力明显提高。认真贯彻计划生育基本国策，人口自然增长率为3.24‰。全民健身活动蓬勃开展，承办多次国内外重大体育赛事。推进新型社区建设，社区服务功能进一步完善。

　　人防、新闻出版、广播电视、民族宗教、外事侨务、国家安全、对台、老龄、残联、武装、档案、地方志等工作取得新成绩。

　　民主法制和精神文明建设进一步加强。自觉接受人大依法监督和政协民主监督，办复人大代表建议552件，政协委员提案922件，办复率100%。坚持依法治市，完善规章制度，起草审核8件地方性法规草案，制定26件规章和22件规范性文件。建立区、县（市）法律援助中心，办理法律援助案件1568件。依法维护农民工权益，清理拖欠农民工工资1.24亿元。安全工作进一步加强，事故发生次数和死亡人数分别下降7.46%和8.33%。推进依法信访，拓宽为群众服务渠道。开展创建“平安哈尔滨”活动，刑事案件综合发案率下降5.5%。继续打击“法轮功”等邪教组织。实施《哈尔滨市2004-2007年创建文明城市工作规划》，深入开展“树立哈尔滨形象，创建文明城市”和“信守社会公德、争做文明市民”活动，促进了全民素质的提高。加强和改进未成年人思想道德建设，初步形成学校、社会、家庭“三位一体”的育人体系。

　　（七）着力提高行政效能，大力优化经济发展环境

　　认真贯彻《行政许可法》和国务院《全面推进依法行政实施纲要》，积极推进行政效能建设，不断提高行政能力，优化经济发展环境。制定了《哈尔滨市行政机关工作人员七条禁令》，从严整治行政“不作为”和“乱作为”，提高了办事效率和公共服务质量。组建市政府行政执法监督局，加强行政执法监督。推进行政审批制度改革，调整、取消行政许可事项201项。成立市行政审批中心，45个部门集中办公，实行“一站式”办理、“一条龙”服务、“一个窗口”收费，受理审批服务事项3.8万件，办结率98.9%，投诉率为零。推进政务公开，“中国·哈尔滨”政府门户网站日趋完善，排名居全国前列，38个政府部门在网站公开政务事项，13个部门与市民开展网上对话，21个部门提供了办事指南。坚持“课题制”的方法，解决经济社会发展中的一些热点、难点问题，推动了政府工作创新。完成新一轮政府机构改革，乡镇机构改革基本结束。

　　各位代表，过去的一年，是哈尔滨在振兴道路上奋力前进的一年，也是老工业基地潜能得到发挥、比较优势进一步显现的一年。多年成果的积累，我市综合实力进入全国城市前10位，更加坚定了我们振兴哈尔滨的信心；实施振兴老工业基地战略，我市受到国内外关注，大开放的趋势越来越明显；国有企业改革、解决“三农”问题取得较大进展，为全面突破体制性、结构性障碍奠定了基础，积累了经验；行政区划调整，市行政中心迁移松北，使城市发展空间得到极大扩展，为哈尔滨都市经济圈的形成构建了基本框架；全市上下求发展、谋振兴的合力进一步形成，干事创业的气氛更加浓厚。这种大好局面来之不易，是党中央、国务院和省委、省政府及市委正确领导的结果，是市人大、市政协、民主党派、工商联和群众团体有力支持和监督的结果，是全市人民团结一致、奋力拼搏的结果。在此，我代表市人民政府向全市各族人民，向驻哈部队和武警官兵，向国家和省驻哈单位，向港澳台同胞、海外侨胞及国际友人，向所有关心、支持、参与哈尔滨建设的同志们、朋友们，致以衷心的感谢和崇高的敬意！

　　我们清醒地认识到，当前经济社会发展中还存在许多困难和问题。投入和对外贸易对经济发展拉动力不强，加快发展动力不足；国有企业改革进入最后攻坚阶段，全市还有 1000多户国有企业尚未改制，今年将面临改制成本进一步加大的压力；支柱产业竞争力较弱，在我国“入世”保护期即将结束的形势下，汽车、医药等重点行业将遇到新的更大的市场冲击；农业和农村经济受自然因素、市场因素影响大，持续发展的基础还比较脆弱，保持去年的发展势头将十分困难；社会保障体系不尽完善，保障能力仍然不强；民营经济发展缓慢，有实力的民营企业集团和大规模的民间投资还不多；招商引资水平不高，具有牵动作用的生产性大项目比较少；城市规划、建设、管理科学水平有待进一步提高；政府职能转变还不到位，行政效能建设有待进一步加强，发展环境需要继续改善。解决这些矛盾和问题，将是对政府行政能力的严峻考验。

　　二、以科学发展观为指导，推动经济和社会事业快发展、大发展

　　在我们刚刚跨进新的一年之际，我市成功获得了2009年第24届世界大学生冬季运动会主办权，不仅坚定了我们继续申办冬奥会的信心，也为哈尔滨快发展、大发展提供了又一次重要机遇。未来几年，我们将以筹办大冬会为契机，推动全市经济、文化、体育、教育和环境的全面发展，促进全民素质迅速提高。2005年是“十五”计划决胜年，也是振兴老工业基地的关键之年。我们要以邓小平理论和“三个代表”重要思想为指导，深入贯彻党的十六大、十六届三中、四中全会、中央经济工作会议、省委九届六次全会、市委十一届三次全会精神，全面树立和落实科学发展观，以振兴老工业基地为中心，坚持“加快发展、当好龙头”的战略部署和“努力快发展，全面建小康，振兴哈尔滨”的工作主题，着力推进改革开放，加快经济结构调整，转变经济增长方式，进一步提升城市综合竞争能力；加强政治文明和精神文明建设，维护社会稳定，构建和谐社会，努力实现经济社会全面协调可持续发展，为启动“十一五”规划奠定坚实基础。

　　主要预期目标是：全市生产总值增长12%，其中第一、二、三产业分别增长6%、15%、12%；全社会固定资产投资增长20%；地方财政一般预算收入增长9%；社会消费品零售总额增长12%；市属企业进出口总额增长20%；实际直接利用外资增长20%；城市居民人均可支配收入增长8%，农村居民人均纯收入增长5%；城镇登记失业率控制在5%以内；人口自然增长率不超过4.8‰；主要污染物削减2.1%。

　　（一）以国有企业改革为重点，推进体制、机制创新

　　今年是改革任务极其繁重的一年。我们将进一步加大力度,确保各项改革取得重大进展。

　　基本完成国有企业改革。坚持开放式改制，引进战略投资和民营资本参与国有企业改革，力争90%以上的国有企业进入改制程序，70%的企业基本完成改制。针对企业不同情况，实行一企一策。对规模大、影响力强的企业，引进战略投资者。对处于盈亏边缘但有发展前景的企业，推进混合所有制改革。对停产或半停产、产品或部分产品有市场的企业，采取整体出售等多种措施。对劣势企业实施破产关闭。同步推进配套改革。完成22所企办校分离工作，分离10所企办医院，启动企办供电、供水、供热和物业部门的分离工作。积极与国家政策互动，推动国有大中型企业主辅分离、改制分流工作。推进企业债务重组，力求在回购企业不良贷款方面取得实质性进展。进一步深化国有资产管理体制改革，完善授权经营制度，创新监管机制，逐步建立符合市场经济要求的国有资产监管和运营体系。

　　完成事业单位改革的阶段性目标。进一步整合资源，优化布局，重点抓好教育、文化、卫生、城建、城管等行业改革，全面完成人员聘用工作，实现用人机制转换，建立长效管理机制。加快推进后勤服务社会化，按照“养事不养人”的原则，最大限度发挥财政投入效能，探索多元化投入办事业的途径。积极推进事业单位产权制度改革。改革事业单位分配制度，逐步建立吸引人才、激励人才、留住人才的分配机制，调动职工的积极性和创造性。

　　继续深化农村改革。巩固农村税费改革和乡镇机构改革成果，坚决制止和杜绝一切乱收费现象，进一步减轻农民负担。充分利用土地资源，采取多种有效方式推动农村土地流转和适度规模经营。深化粮食流通体制改革，完善对农民的直接补贴制度。加快农村信用社改革，扩大县域企业贷款担保范围，推广农民小额信贷。继续推进农村新型合作经济组织试点工作。保持农村社会稳定，解决好土地承包纠纷等农村出现的新情况、新问题。加快推进农村社保并轨工作。

　　加快民营经济发展。出台《加快民营经济发展的若干意见》，消除制约民营经济发展的体制性障碍，努力营造促进民营企业加快发展的政策环境。以国有中小企业民营化为方向，鼓励民营企业参与老工业基地调整改造。完善民间投资机制，引导民间资金向民间资本转化。建立健全服务体系，帮助民营企业解决资金、信息等生产经营中的困难和问题。帮助符合条件的民营企业进入资本市场，扶持一批民营科技企业进入“小巨人”行列。

　　深化行政管理体制改革。基本完成集体企业改革。做好国有商业企业改革的后续工作。推进投融资体制改革。加快城市供热体制改革。

　　（二）加快调整改造步伐，推进新型工业基地建设

　　积极参与哈大齐工业走廊建设。加快工业重大项目建设，积极培育优势产业、强势集团和知名品牌。实现工业增加值580亿元，增长15%以上，力争达到600亿元。规模以上工业利税增长10%，利润增长12%。

　　扩大投资规模，实施重大项目建设。落实好前两批国债项目，规划论证好第三、四批国债项目，争取国家和省的更大支持。完成工业投资140亿元，增长20%以上，实施超千万元以上项目234项，确保竣工投产70项以上。重点实施33个超亿元项目。推进超大规格铝板带材、轴承系列产品、精密数控刀具等一批重大改造项目。

　　壮大优势产业集群。加快发展机械、医药、食品等优势产业，延伸产业链条，推进产业聚集。机械工业做强做大主机产业，加快发展配套产业，力争新开59个项目，投产26个项目，增加配套企业30户，使配套率增长20％以上。医药工业新开25个项目，投产15个项目。食品工业新开10个项目，投产13个项目。优势产业在建和新建企业中，力争引进10户以上国际大企业。加快培育新的优势产业。规划、启动化工产业园区建设，推进12个化工重大项目，以哈石化为龙头，构建产业链，力争2—3年把化工产业培育成我市新的优势产业。以提高资源利用效率为目标，大力发展循环经济和节约型经济，推动符合国家产业政策、科技含量高、符合环保要求的新兴产业发展，加快锂离子电动车、直线电机、多用途轻型飞机等产品的产业化步伐。依托资源优势和现有产业基础，发展木材加工业。

　　培育大企业集团，增强核心竞争力。加强政产学研结合，加快培育和发展企业技术中心，致力研制具有自主知识产权的产品，加快产业化步伐。发挥哈药、哈啤、电站、哈飞等品牌优势，通过资源整合、多元化投资，建设一批品牌号召力强、技术创新能力强、市场应变能力强的大企业集团。

　　（三）深入解决“三农”问题，推动县域经济持续快速发展

　　坚决贯彻落实中央1号文件精神，巩固和发展农村经济大好局面。围绕农民和县（市）财政双增收目标，继续完善政策、创新体制、优化结构，推动县域工业化、农业产业化、农业科技化、农村城镇化，巩固粮食生产能力，提高农业综合效益，继续促进农民增收。

　　推动农业结构优化。重点发展粮食、食品加工、畜牧业、绿色和无公害食品、林业、“北药”六大产业。完善农产品标准化、质量安全和认证体系，扩大绿色、无公害食品种植面积，培育一批有机食品基地。扩大经济作物、饲料作物种植面积。继续推进粮牧“主辅换位”，加快发展畜牧业，推广专业化、规模化和集约化养殖。以棚室蔬菜和花卉为重点，发展城郊型农业。

　　推动农产品流通体系建设。培育粮食、供销、外经贸企业等农产品流通市场主体。发展农产品流通大户和农村经纪人队伍，引导各类专业协会、专业合作社等农村新型合作经济组织发展。加强粮食、蔬菜、畜禽产品、山产品、绿色食品等专业批发市场建设。加大农业生产信息化建设力度，加强与农产品销区合作。大力发展订单农业。

　　发展龙头在城镇、基地在农村的城乡互动型产业。推进城乡统筹规划、资源统一配置、产业相互衔接。引导县域企业加入大企业分工协作体系，发展配套产业。加快园区建设，构建承接城市产业转移和招商引资的平台。通过引进、合作等方式，建立一批大型农产品加工企业，推动农业产业化。

　　加快农村劳动力转移。加强农民工专业化、规范化培训，提高农民工就业技能。加强农村劳动力转移的组织和管理，畅通劳动力供求信息渠道，促进劳务输出。改善农民进城就业环境，依法维护进城就业农民权益。加快农村城镇化，发展中心镇，扶持小康示范镇，培植立镇产业，为劳动力转移提供载体。

　　推动农业基础设施建设，搞好土地资源合理开发利用。加大对农业的投入力度，推进农田水利、小流域综合治理和县乡村道路建设。继续做好退耕还林、还湿、还草工作。搞好农业综合开发和扶贫解困，继续实施革命老区饮水工程，入户率达到80%。科学规划和安排建设用地。做好征用土地补偿工作，保护失地农民利益。依法促进土地流转，支持土地适度规模经营。

　　（四）推动服务业优化升级，完善现代城市服务功能

　　大力发展旅游业。以冰雪品牌为龙头，努力把冰雪游、金源文化游、欧陆风情游、森林生态游做大、做强、做精。打造“清凉世界”夏季旅游品牌，开发农业生态观光游、工业游、科技游等新领域，实现旅游产品多样化、四季有热点。提高旅游业文化含量，提升旅游活动品位。搞好市场化运作，引导更多企业投资开发旅游产业。加强旅游业法制化建设和规范化管理，提高旅游服务质量，改善旅游环境。

　　加强金融和财税工作。建立政府、企业、银行间的协调机制，进一步发挥金融在实施振兴老工业基地战略中的作用。推进财税体制改革和财源建设，整顿财税秩序。严格税收征管，强化预算约束，努力增收节支。深化财政支出体制改革，提高财政资金使用效益，推动公共财政建设。

　　推进商贸服务业发展。继续推进服务业市场开放，提升服务业集聚外部资源的能力和接轨国际市场的程度。编制《哈尔滨市商品市场体系建设规划》，引导企业合理布局、适度集聚、公平竞争。加强对社区服务业的引导和政策扶持，通过社会参与、市场化运作方式，建立和完善贴近城乡居民生活的社区服务业网络,增强服务业便利民生、吸纳就业、稳定社会的功能。

　　培育物流、咨询、信息、科技、文化等现代服务业，发展总部经济、假日经济、会展经济、知识经济等新经济产业。

　　（五）突出对俄经贸科技合作，进一步提高招商引资和对外开放水平

　　落实省委、省政府要求，全力推进对俄经贸科技合作战略升级。搞好对俄科技项目规划和对接，加强与俄在关键核心技术方面的合作。以科研合作、教育培训和信息交流为重点，开发拳头产品，培植规模企业，吸引优秀人才，建立创新体系，把哈尔滨建成全国对俄科技合作基地。以俄罗斯市场为导向，发挥我市产业优势，加强与国内外知名企业合作，建设对俄出口加工基地和物流集散中心，建立对俄经贸直销通道。依托我市技术和劳动力优势，积极参与对俄资源开发。

　　全面做好外经外贸工作。加大多元化市场开发力度，调整、优化进出口商品结构，高起点引进技术和设备，提高出口产品技术含量，努力扩大对外贸易规模和质量。鼓励和支持有比较优势的企业“走出去”，开展境外加工贸易、对外承包工程和劳务输出，加强能源、原材料开发合作，在更大范围、更广领域、更高层次参与国际经济技术合作。

　　继续推进开发区建设。强化产业政策导向，进一步整合资源，完善开发区载体功能，加快各类工业园区建设，发展产业链和产业集群。进一步优化投资环境，加快技术创新体系建设，提升开发区项目承载能力和环境竞争力。

　　大力开展招商引资工作，努力提高利用外资规模和质量。认真做好招商引资专项规划，搞好项目储备和推介。积极参与重大招商、经贸活动。吸引外商参与我市产业结构调整、国企改革和东北亚区域合作。鼓励外商在我市建立研发基地、生产基地和区域性营运管理基地。大力开展重点地区、重点园区、大型企业和重点项目招商。创新招商方式，拓宽招商引资渠道。建设招商引资网络平台，开展网上招商、委托招商和中介招商。进一步把“哈洽会”办出特色，带动会展经济发展。实行诚信招商，搞好签约项目落实和落户项目服务，提高合同履约率。加强与国内较发达地区和东北各城市的交流与合作。

　　（六）突出城市特色，全面提升城市规划、建设、管理水平

　　以“大气、洋气、灵气”为目标，提高城市规划的国际化水平。按照振兴老工业基地战略需要，规划以市区为中心，以100公里左右为半径的1小时都市经济圈。提高城市规划整体性，科学规划城区核心经济圈，完成重点地段城市设计和控详规划、松北分区规划、群力新区基础设施专项规划的编制工作。做好主城区产业基地规划，形成以优势产业、对俄经贸科技合作为主的产业带。按照建设生态型园林城市的需要，规划一批生态廊道和风景区。

　　功能建设和文化延展并重，提高城市建设现代化水平。实施一批路桥建设项目，完善城市路网体系。力争启动轨道交通一期工程，确保实施一项跨松花江大桥或隧道工程。抓好事关人民群众生产生活的重点路桥建设，打通通乡街，拓宽改造工农大街。加快推进基础设施建设。集中力量推进磨盘山供水工程，确保2006年向城市供水。加强地下基础设施管理，加快城市供热、供水、排水、燃气等管网建设和改造。启动何家沟综合整治、文昌污水处理三期等内河治理和污水处理项目。继续实施道里集中供热二期、西南部集中供热、向阳生活垃圾处理场二期工程。完成西南部生活垃圾无害化处理厂一期工程。加快园林绿化建设。继续推进城区增绿、郊区环绿，对沿松花江、穿城铁路线及城区“三沟”进行绿化，提高城市绿化覆盖率。加强历史文化名城保护工作，继续推进特色文化街区建设。重点实施中央大街综合整治三期后续工程、太阳岛三期改造、北方森林动物园二期、俄罗斯风情园改造、印度风情街二期改造建设工程，进一步彰显城市文化特色和品位。继续推进住宅建设。严格控制高档商品房，大力发展普通商品房，适量建设经济适用房。推进“城中村”开发改造。继续把环境综合整治向贴近群众生活领域延伸。着力解决人民群众行路难、过街难和入厕难问题，建设和改造一批背街背巷、无灯街路、破损人行道、水冲公厕和居民庭院，力争消除现有市政土路。

　　加大依法管理城市力度，提高城市管理精细化水平。坚持人性化管理和可持续发展的原则，深化城市管理体制改革，完善市、区、街三级管理网络，推进城市管理重心下移。加快城市管理法规建设，加强城市管理综合执法，继续扩大综合执法范围，努力消除城市管理的盲区和空白点。完善监督管理体系，实现事后处理整改向超前预防控制转变。推动城市管理信息化。强化城市交通管理，树立以人为本、公交先行的交通管理理念，建立相应的交通组织管理模式。努力避免城建施工给群众生活带来不便。研究制定符合我市地域特点的清冰雪机制。

　　搞好经营城市工作，提高城市建管的市场化水平。深化城建投融资体制改革，发挥城投公司融资平台作用，建立良性偿债机制，探索融资新途径。采取BOT、TOT、合资合作等多种方式，吸引外资和社会资本参与大项目建设。推行土地收购储备和招标拍卖制度，发挥土地资产效益。加快市政公用事业改革，推进经营性市政公用设施建设和运营市场化。实行特许经营制度试点，把清扫保洁、道路维修养护、绿化抚育等推向市场。

　　（七）坚持以人为本，推进和谐社会建设

　　努力做好减贫扶弱和社会保障工作。千方百计扩大就业再就业。进一步落实财政投入、小额贷款、税收减免、社保补贴、岗位补贴、培训补贴等政策，发展劳动密集型加工业、第三产业和社区服务业，发展民营经济、劳务经济，努力扩大就业容量，全年增加10万个就业岗位。继续实施再就业援助行动，安置1.5万名“4050”人员实现再就业。推进国有企业下岗职工基本生活保障向失业保险并轨，争取国家政策支持，积极筹措地方匹配资金。建立依法参加社会保险机制，扩大社会保险覆盖面。养老保险突破100万人，新增参保人员10万人，把个人养老账户做实6个百分点，解决近10年来个人养老账户空账问题。改革养老金计发办法，确保养老保险金全部按时足额发放。失业保险达到176万人，基本实现全覆盖。完善多层次医疗保险体系，医疗保险力争达到155万人，比上年增加12万人。继续提高城市低保金补差额，开展农村低保工作。建立和完善城市低收入家庭供热保障制度。深入开展“慈善济困民德工程”、“扶贫结对”等活动，巩固发展“慈善超市”，建立直接、便捷、可靠的救助通道。改造城区危棚房80万平方米，提供380套廉租住房，力争全部解决人均住房使用面积不足6平方米的贫困户住房问题。深入实施“扶贫开发整村推进战略”。

　　加强社会主义精神文明建设。贯彻落实《公民道德建设实施纲要》，围绕提高全民思想道德、科学文化和健康素质，开展群众性精神文明创建活动，培育“四有公民”，促进人的全面发展。以争创文明城市为目标，深入开展“树立哈尔滨形象”和“信守社会公德、争做文明市民”活动；以建设“诚信哈尔滨”为目标，开展“共铸诚信”活动；以提高未成年人综合素质为目标，完善学校、社会、家庭“三位一体”育人网络，强化未成年人思想道德教育。

　　做好社会稳定工作。深入开展创建“平安哈尔滨”活动，推进社会治安综合治理，维护社会稳定和国家安全。坚持专群结合，防范、控制、打击、整治并举，依法惩治各类犯罪活动。坚决打击境内外敌对势力、“法轮功”等邪教组织及“黄、赌、毒”等社会丑恶现象。建立安全长效管理机制，健全灾害预防和应急管理机制，加大安全检查、监督力度，防止重特大事故发生。高度负责地做好群众来信来访工作，引导群众依法信访，遏制越级访、集体访、异常访和重复访的发生。

　　（八）统筹经济社会发展，促进各项社会事业全面进步

　　做好科技和人才工作。发挥科技资源优势，加快科技成果转化。建立科技创新体系，加大对重大项目和重点企业支持力度，启动8个工程技术研究中心和20个实验室建设，推进全液压起重机、畜禽疫苗等10个重点科技攻关项目。加快科技园区建设，增强辐射能力，形成高科技企业、高等院校、科研机构的聚集效应。优化市场配置人才的政策环境和服务环境。大力开发人才资源，加强专家队伍建设，引进国外智力，加强博士后工作站和留学人员创业园建设，培育技能型人才，为振兴老工业基地提供人才智力支持。

　　继续推进数字化城市建设。以公共服务为重点，加快政务信息网络建设，做好社区信息化试点推广工作。以信息化推动工业化，提高企业现代化管理水平。加强网络和信息安全工作。加快网上审批系统、城市地理信息公共服务平台、企业基础信息共享系统、金卡工程、电子缴税入库系统、人口信息交换平台等应用系统的开发、推广和利用。

　　加强文化建设。制定并实施《哈尔滨文化兴市总体规划》。推进文化体制和机制创新，完善文化产业政策，提高文化产业竞争力。整合文化产业资源，逐步形成由市场配置文化资源的机制。加强文化设施和文化市场管理，打造文化精品工程，普及群众文化，丰富社区文化、企业文化、校园文化和广场文化。坚持正确舆论导向，发展社会科学、文艺创作、新闻出版、广播电视等各项事业。积极发展对外文化交流与合作，展示哈尔滨的文化内涵和魅力。

　　做好教育工作。巩固基础教育，发展高等教育，实行全民终身教育。进一步完善“以县为主”的农村义务教育管理体制，提升农村教育水平。发展职业技术教育，使职业教育与市场需求相适应。加快优质高中建设，扩大优质高中教学资源。改善薄弱学校，改革办学形式，建立教育人才流动机制，提高办学质量。改造193所农村学校危房。

　　加强环境保护工作。开展环保模范城区、生态示范县（市）创建工作。转变经济增长方式，从生产、流通、消费、排放四个环节，培育一批循环经济示范企业。以环境容量为基础，实行污染物排放总量控制，严格实施环境与发展综合决策、环境影响评价制度和排污许可证制度。治理烟尘、扬尘、流域和养殖污染源500个。加快市区环城生态功能圈等18处生态功能保护区建设。创建绿色乡镇、社区、学校、企业200家。

　　做好卫生、体育、人口等项工作。加强公共卫生管理，建设突发性公共卫生事件管理体系，完善疾病预防控制体系和卫生监督执法体系。继续实施食品药品放心工程，建立和完善食品市场准入制度，加强药品供应网、监督网建设。做好非典、禽流感、艾滋病等重大传染病的防治工作。扩大农村新型合作医疗试点范围，加快农村乡镇卫生院建设。普及全民健身活动。启动大冬会筹备工作，搞好比赛场馆设施规划、建设和改造，提高冬季运动项目竞技水平。落实计划生育基本国策，完善人口与计划生育公共服务体系，稳定低生育水平。实施妇女儿童发展规划。做好第一次经济普查工作。推进审计、外事侨务、对台、民族宗教、老龄、残联、武装、人防、气象、档案、地方志等工作。

　　（九）加强效能建设，提高行政能力

　　按照党的十六届四中全会提出的“加强党的执政能力建设”的要求，以提高行政能力为重点，努力建设服务政府、责任政府、法治政府、廉洁政府。

　　推进依法行政，努力改善发展环境。贯彻人大及其常委会决议，完善工作报告制度，自觉接受人大的依法监督、政协的民主监督和人民群众的社会监督。强化法治观念，严格依照法定权限和法定程序行使权力、履行职责，做到依法决策、依法办事、依法处理问题，把政府各项行政行为纳入法制化轨道。贯彻《行政许可法》，深化行政审批制度改革，建立行政许可配套制度，加强对行政机关实施行政许可的有效监督。树立公平和效率并重的理念，切实履行经济调节、市场监管、社会管理和公共服务职能，转变政府调节经济的方式，真正把经济管理职能转到主要为市场主体服务和创造良好发展环境上来。探索相对集中行政许可的有效途径和措施，进一步减少审批事项和程序。继续推进行政机关效能建设，建立健全行政效能考核、评估机制，加大监督检查力度，努力提高服务效率和质量。坚决整治“三乱”，突出解决好工程验收问题，严肃处理勒卡、刁难企业的单位和个人。加强行政复议、行政执法投诉工作。

　　完善政府决策机制，提高科学化、民主化决策水平。坚持实行“课题制”，完善公众参与、专家论证、政府决策相结合的决策机制。建立健全重大事项集体决策制度、人民建议征集制度、专家咨询制度、社会公示和社会听证制度；建立健全决策信息反馈、决策后评估、决策失误纠错改正机制；建立健全决策监督和责任追究制度，按照“谁决策、谁负责”的原则，对超越权限、违反程序决策造成重大损失的，追究决策者责任。提高政务工作透明度，促进政府与市民行为的互动。

　　切实转变作风，狠抓工作落实。把实现群众愿望、满足群众需要、维护群众利益，作为政府工作的出发点和落脚点。求真务实，注重实效，把工作着力点放到研究解决改革发展稳定中的重大问题上，放到研究解决群众生产生活中的紧迫问题上。进一步精简会议，压缩文件，改进会风、文风。完善干部政绩考核体系，建立严格的目标责任制和奖惩制度，形成抓落实的工作机制。

　　坚持从严治政，切实加强廉政建设和反腐败工作。以开展保持共产党员先进性教育为统领，深入开展“两风”建设，有效开展警示教育。建立健全教育、制度、监督并重的惩治和预防腐败体系，推动反腐倡廉工作向纵深发展。抓好领导干部廉洁自律，加大行政监察、审计监督力度。健全和完善政府内部反腐倡廉的监督制约机制，努力提高公务员队伍廉洁自律的自觉性，增强与腐败行为作斗争的坚定性。坚决克服官僚主义和形式主义。加大对大案要案查处力度，坚决制止损害群众利益的行为。

　　各位代表，实现哈尔滨的全面振兴任重道远。我们要更加紧密地团结在以胡锦涛同志为总书记的党中央周围，高举邓小平理论伟大旗帜，以“三个代表”重要思想为指导，在省委、省政府和市委的领导下，发挥全市人民的积极性和创造性，真抓实干，迎难而上，推动哈尔滨快发展、大发展，为实现全面建设小康社会的宏伟目标而努力奋斗！
